# Reaction mechanism of hydrogen-tritium exchange reactions between several organic and HTO molecules: a role of the second HTO

**DOI:** 10.1039/c7ra13110k

**Published:** 2018-01-22

**Authors:** Taro Udagawa, Masanori Tachikawa

**Affiliations:** Department of Chemistry and Biomolecular Science, Faculty of Engineering, Gifu University Yanagido 1-1 Gifu 501-1193 Japan udagawa@gifu-u.ac.jp; Quantum Chemistry Division, Graduate School of Science, Yokohama City University Seto 22-2, Kanazawa-ku Yokohama 236-0027 Japan

## Abstract

The mechanism of hydrogen-tritium (H-T) exchange reactions between several small organic and HTO molecules have been investigated using M06-2X/6-311++G(d,p) method. The second HTO molecule is taken into account for both direct and addition–elimination H-T exchange reactions. The reactivity of small organic molecules for H-T exchange reactions is in the order of CH_3_COOH > CH_3_CH_2_OH > CH_3_CHO ≈ CH_3_COCH_3_ ≈ C_2_H_4_ and C_3_H_6_ > CH_4_, C_2_H_6_, and C_3_H_8_. In particular, the energies of activation in addition–elimination H-T exchange reactions of alkene with two HTO molecules become lower than those of direct H-T exchange ones. Our study reveals that (i) the reactivity of alkene with HTO molecules is comparable to that of aldehyde and ketone when the effect of the second HTO molecule is taken into account and (ii) the H-T exchange reactions between alkene and HTO molecules prefer addition–elimination H-T exchange mechanism, whereas other organic molecules favor a direct one.

## Abstract 2

The mechanism of hydrogen-tritium (H-T) exchange reactions between several small organic and HTO molecules have been investigated using M06-2X/6-311++G(d,p) method.

## Graphical abstract

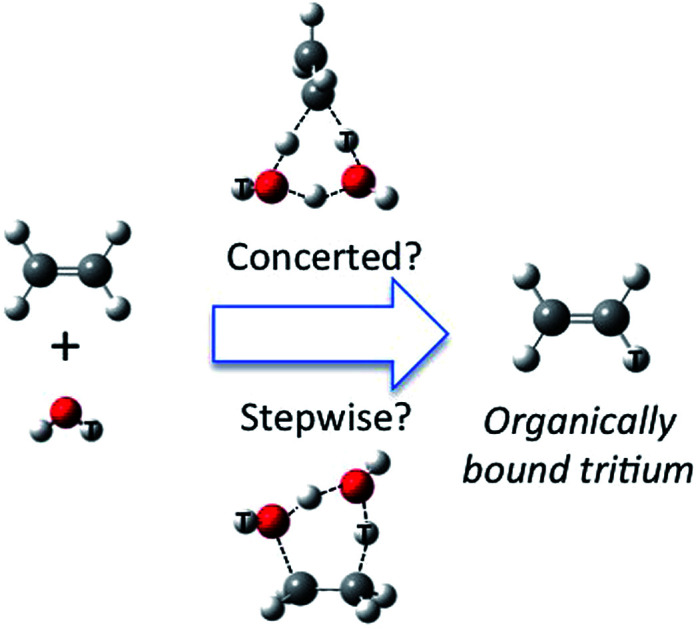

## Introduction

Tritiated water, HTO molecule, is known as a toxic and radioactive molecule and is considered as one of the components of tritium-containing waste oils.^1^ Although the amount of tritium is pretty small in nature, a large amount of tritium-containing oil is generated in tritium-related facilities.^2^ Since tritium-containing waste oil also contains soluble tritium-exchangeable organic molecules, HTO molecules easily react with them to produce highly toxic organically bound tritium (OBT). Therefore, many research groups have earnestly studied this issue.^2–7^ Especially, developing a method to extract tritium from these tritium-containing wastes and understanding the reactivity of tritium-containing species, such as HTO molecules and OBT, are the prime tasks.

Since these tritium-contaminated species are toxic as mentioned above, the experimental treatment will be difficult and theoretical approaches are quite useful to investigate the reactivity of these compounds. Despite the validity of theoretical approaches, only a few theoretical researches have been published so far.^5^ Quite recently, Dong and coworkers studied hydrogen-tritium (H-T) exchange reactions between small organic and T_2_ (ref. 8) or HTO^5^ molecules using density functional theory (DFT). As the more reliable exchange–correlation density functionals have been proposed,^9–13^ DFT becomes the most familiar quantum mechanics (QM) tool for not only theoretical chemists but also experimental chemists, nowadays. Theoretical investigation using reliable QM method, such as DFT, is indispensable to understand the reaction mechanisms in detail. Dong and coworkers studied H-T exchange reactions between small organic and HTO molecules using M06-2X DFT method.^5^ They calculated 17 direct H-T exchange reactions and 11 addition–elimination H-T exchange reactions, and obtained following conclusions: (1) H-T exchange reactions between organic and HTO molecules could proceed with direct H-T exchange mechanism or addition–elimination H-T exchange mechanism. Direct H-T exchange mechanism is kinetically favored for the all reactions studied, (2) the direct H-T exchange reactions occur *via* less strained six-membered ring transition state (TS) structures with the aid of the second HTO molecule, O–H group, C=O group, or COOH group and (3) the reactivity of small organic molecules for H-T exchange reactions with HTO molecules is in the order of carboxylic acid > alcohol > aldehyde ≈ ketone > alkene > alkane.^5^

Although Dong and coworkers adequately took into account the effect of the second HTO molecule for direct H-T exchange reactions, they took only one HTO molecule for addition–elimination H-T exchange reactions *via* high-strained four-membered ring TS. Thus, we believe that it is indispensable to take account of the effect of the second HTO molecule on addition–elimination H-T exchange reactions, to adequately understand the mechanisms of the H-T exchange reactions. We, therefore, would like to investigate H-T exchange reactions between small organic molecules and the adequate number of HTO molecules using M06-2X DFT method. In this study we especially focus on the role of the second HTO molecule for addition–elimination H-T exchange reactions.

## Computational detail

We have investigated the direct H-T exchange reactions between following organic molecules and one or two HTO molecules: (D-(a)) CH_4_, (D-(b)) C_2_H_6_, (D-(c)) C_3_H_8_, (D-(d)) C_2_H_4_, (D-(e)) C_3_H_6_, (D-(f)) CH_3_CHO, (D-(g)) CH_3_COCH_3_, (D-(h)) CH_3_CH_2_OH, and (D-(i)) CH_3_COOH. According to the Dong's previous study,^5^ only six-membered TS structures are considered for these reactions. For the reactions D-(f), D-(g), and D-(i), only one HTO molecule is needed to form a six-membered ring TS structure by participation of C=O or COOH group, otherwise the second HTO molecule is required.

We have also investigated the addition–elimination H-T exchange reactions between following several organic and HTO molecules: (AE-(a)) C_2_H_4_, (AE-(b)) C_3_H_6_, (AE-(c)) CH_3_CHO, (AE-(d)) CH_3_COCH_3_, and (AE-(e)) CH_3_COOH. Only the reactions with one HTO molecule were investigated in the Dong's previous study, although such reactions can form only high-strained four-membered ring TS structures. In the present study, both the reactions with one and two HTO molecules were investigated to clearly demonstrate the role of the second HTO molecule on addition–elimination reactions.

All the stationary point structures of these reactions were optimized by M06-2X DFT method with 6-311++G(d,p) basis set, and normal mode analyses were also performed to characterize these optimized structures. We confirmed that all structures have the appropriate number of imaginary frequencies; 0 for minimum and 1 for TS structures. For the following discussion, the harmonic zero-point vibrational energy (ZPVE)-corrected energies (*E*), enthalpies (*H*), and Gibbs free energies (*G*) computed at *T* = 298.15 K and *P* = 1 atm were used. All calculations were performed with the aid of GAUSSIAN 09 program.^14^

## Results and discussion

### Direct H-T exchange reactions

**Fig. 1:**
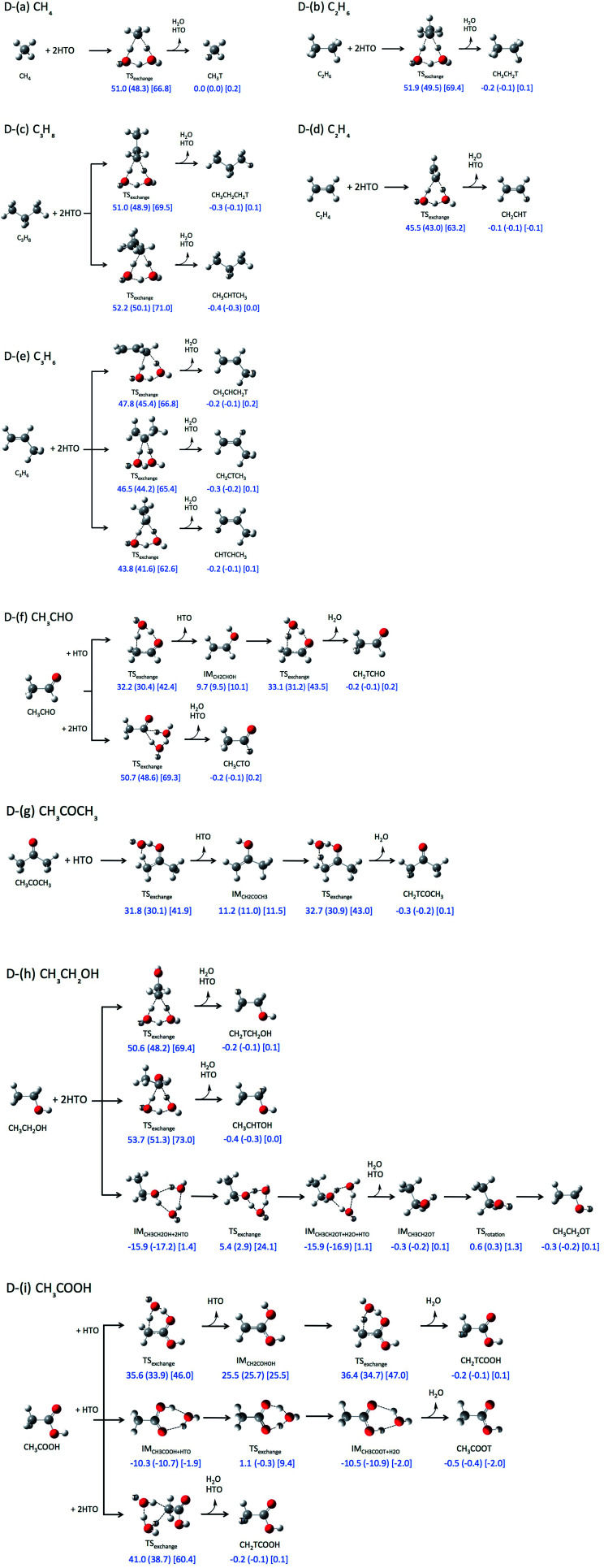
Reaction pathways and relative zero-point vibrational corrected energies [kcal mol^−1^], enthalpies [kcal mol^−1^] (shown in round bracket), and Gibbs free energies [kcal mol^−1^] (in square bracket) of direct H-T exchange reactions obtained by M06-2X/6-311++G(d,p) level of calculations.

First, we investigated direct H-T exchange reactions using M06-2X/6-311++G(d,p) method. The reaction mechanisms and the calculated relative ZPVE-corrected energies (Δ*E*), enthalpies (Δ*H*, shown in round brackets), and Gibbs free energies (Δ*G*, in square bracket) are summarized in Fig. 1. Tritium atoms are depicted by white ball with symbol “T” in Fig. 1. In these direct H-T exchange reactions, one or two HTO molecules participate to form the stable six-membered ring TS structure. If C=O or COOH group can participate, only one HTO molecule is needed to form six-membered ring TS structure, otherwise the second HTO molecule is required for the formation. While there are several conformations for the reactions with two HTO molecules, only the most favorable reactions are analyzed in the present study. In all reactions, hydrogen atom in HTO molecule prefers to act as proton (hydrogen nucleus) donor in hydrogen-bonded interaction rather than tritium atom, since heavier hydrogen isotopes, such as deuterium and tritium, act as weaker hydrogen nucleus donor in hydrogen-bonded interaction.^15^

The ZPVE-corrected energies of activation (Δ*E*^a^), which are corresponding to the relative energies of the TS structures from those of the reactant structures, of the direct H-T exchange reactions between alkane and two HTO molecules (Fig. 1D-(a)–D-(c)) are higher than 50 kcal mol^−1^. Meanwhile, Δ*E*^a^ value of the reaction between alkene and two HTO molecules is 45.5 kcal mol^−1^ for C_2_H_4_ (Fig. 1D-(d)) and 43.8 kcal mol^−1^ for the most favorable reaction of C_3_H_6_ (Fig. 1D-(e)). The reactivity of alkene for H-T exchange reaction with HTO molecules is higher than alkane. The enthalpies of activation (Δ*H*^a^) and Gibbs free energies of activation (Δ*G*^a^) in the reactions between alkene and two HTO molecules are also lower than those in the reactions with alkane. While the Δ*H*^a^ values are similar to the Δ*E*^a^ ones, the Δ*G*^a^ values are about 17 kcal mol^−1^ higher than Δ*E*^a^ ones except for the reaction of CH_4_ (15.8 kcal mol^−1^). Therefore, the entropic contributions are prominent in these direct H-T exchange reactions.

The Δ*E*^a^, Δ*H*^a^, and Δ*G*^a^ values of the reactions with CH_3_CHO (aldehyde), CH_3_COCH_3_ (ketone), and CH_3_COOH (carboxylic acid), in which C=O group can participate to form six-membered ring TS structure, are shown in Fig. 1D-(f), D-(g), and D-(i). The Δ*E*^a^ values for these reactions are 33.1 kcal mol^−1^ (CH_3_CHO), 32.7 kcal mol^−1^ (CH_3_COCH_3_), and 36.4 kcal mol^−1^ (CH_3_COOH), which are clearly lower than the aforementioned Δ*E*^a^ values for the reactions of alkane and alkene. The Δ*H*^a^ and Δ*G*^a^ values in these reactions are also lower than those in the reactions with alkane and alkene. Interestingly, Δ*G*^a^ values are about 10 kcal mol^−1^ higher than Δ*E*^a^ ones in these reactions. Although the second HTO molecule is necessary to form less-strained six-membered ring TS structure for the reactions of alkane and alkene, the increase of the number of molecules in the reaction brings larger entropic contribution. Thus the differences between Δ*G*^a^ and Δ*E*^a^ values are larger in these reaction compared to those in the reactions with one HTO molecule.

The notably small Δ*E*^a^ values are found in the reactions with CH_3_CH_2_OH (Fig. 1D-(h)) and CH_3_COOH (D-(i)), when O–H hydrogen or COOH hydrogen participates to the reaction. The Δ*E*^a^ value of reaction D-(h) is 5.4 kcal mol^−1^, and that of reaction D-(i) is 1.1 kcal mol^−1^, which are prominently smaller than those of other direct H-T exchange reactions.

The above results of Δ*E*^a^ values are basically consistent with the previous Dong's study.^5^ Based on the Δ*E*^a^ values, the reactivity of small organic molecules for H-T exchange reaction with HTO molecules is in the order of CH_3_COOH (carboxylic acid) > CH_3_CH_2_OH (alcohol) > CH_3_CHO (aldehyde) ≈ CH_3_COCH_3_ (ketone) > C_2_H_4_ and C_3_H_6_ (alkene) > CH_4_, C_2_H_6_, and C_3_H_8_ (alkane). It should be noted that although the less-strained six-membered ring TS structure can be formed with the aid of the second HTO molecule, the presence of the second HTO one destabilizes the Δ*G*^a^ values at the same time.

### Addition–elimination H-T exchange reactions

**Fig. 2:**
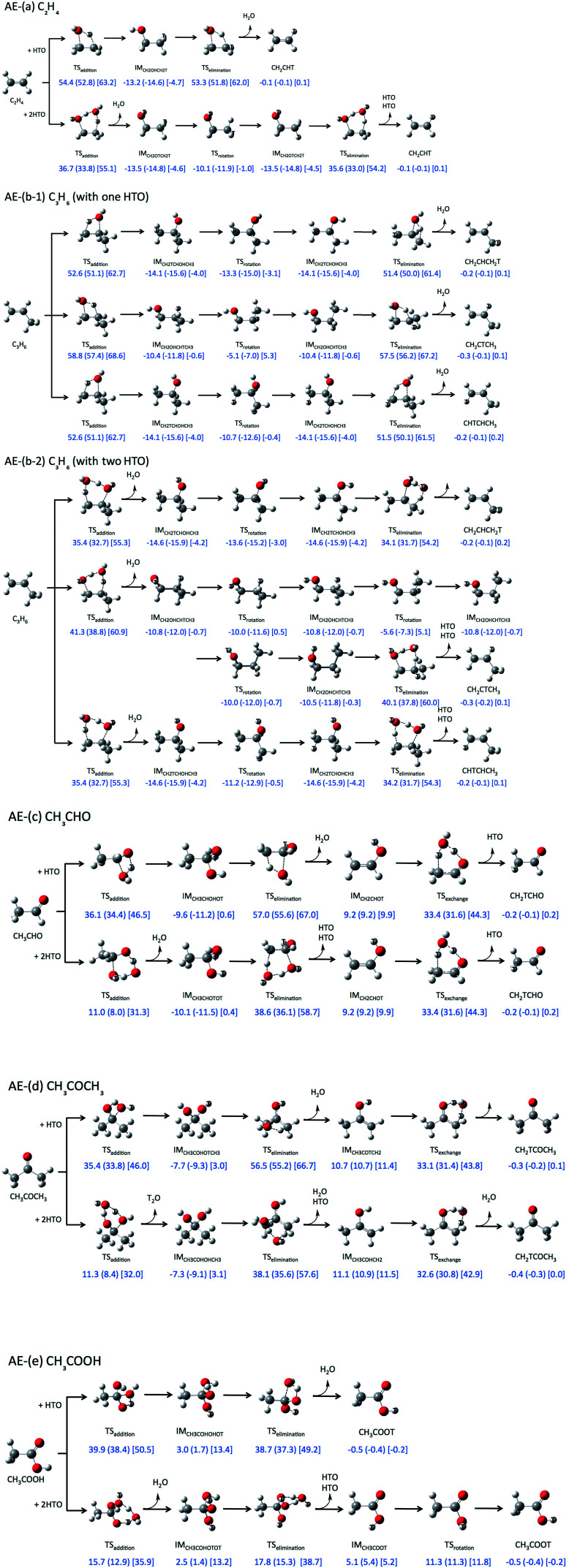
Reaction pathways and relative zero-point vibrational corrected energies [kcal mol^−1^], enthalpies [kcal mol^−1^] (shown in round bracket), and Gibbs free energies [kcal mol^−1^] (in square bracket) of addition–elimination H-T exchange reactions obtained by M06-2X/6-311++G(d,p) level of calculations.

Next, we focus on the addition–elimination H-T exchange reactions. The reaction mechanisms and the calculated relative energies are summarized in Fig. 2. We did not consider the reactions leading to peroxide products in the present study, because these peroxide products are known to be prominently unstable.^5^

First, we would like to focus on the addition–elimination H-T exchange reaction between C_2_H_4_ and one HTO molecule (Fig. 2AE-(a)), following the previous study by Dong.^5^ This reaction is stepwise reaction and has two TSs corresponding to HTO addition process and HTO elimination one. The Δ*E*^a^ value of 54.4 kcal mol^−1^ in the TS of HTO addition is higher than that of 53.3 kcal mol^−1^ of HTO elimination. Such Δ*E*^a^ value is about 9 kcal mol^−1^ higher than that in direct H-T exchange reaction between C_2_H_4_ and two HTO molecules, as shown in Fig. 1D-(d). Meanwhile, the Δ*G*^a^ value of the HTO addition process is 63.2 kcal mol^−1^, which is almost identical with that of the direct H-T exchange reaction in Fig. 1D-(d). Therefore, the direct H-T exchange reaction is no longer preferred in the reaction of C_2_H_4_ when the entropic contributions are taken into account. Fig. 2AE-(a) also shows the calculated relative energies of the reaction between C_2_H_4_ and two HTO molecules. The second HTO molecule lowers the Δ*E*^a^ and the Δ*G*^a^ values of the reaction of C_2_H_4_ + HTO. It is noteworthy that these Δ*E*^a^ and Δ*G*^a^ values (36.7 kcal mol^−1^ and 55.1 kcal mol^−1^) are lower than those of direct H-T exchange mechanism (Fig. 1D-(d), 45.5 kcal mol^−1^ and 63.2 kcal mol^−1^).

Similar entropic contributions and the second HTO molecule effect are also found in the reactions of C_3_H_6_ as shown in Fig. 2AE-(b-1) and AE-(b-2). The Δ*G*^a^ values are 62.7 kcal mol^−1^, 68.6 kcal mol^−1^, and 62.7 kcal mol^−1^ in three addition–elimination H-T exchange reactions of C_3_H_6_ with one HTO molecule (Fig. 2AE-(b-1)), which are almost comparable with those in the corresponding direct H-T exchange reactions with two HTO molecules. Especially, the Δ*G*^a^ value of the addition–elimination reaction towards CH_2_CHCH_2_T with one HTO molecule is lower than that of the direct H-T exchange reaction with two HTO molecules. Taking into account the second HTO molecules, all energies of activation (Δ*E*^a^, Δ*H*^a^, and Δ*G*^a^ values) in the addition–elimination H-T exchange reactions become clearly lower than those in the direct H-T exchange ones, as well as the reactions of C_2_H_4_.

Here, we would like to discuss the anharmonic effect, the solvent effect, and the temperature effect on the H-T exchange reactions of C_2_H_4_ with two HTO molecules. Table 1 shows the relative energies of each stationary point structure corrected for harmonic and anharmonic ZPVE. The anharmonic correction did not provide the significant effect on the relative energy of local minimum structures, since the differences between harmonic and anharmonic values of IM and product are smaller than 0.3 kcal mol^−1^. On the other hand, the anharmonic correction slightly raised the relative energies of TSs. For example, the anharmonic relative energies of TS_exchange_ in the direct H-T exchange reaction and TS_addition_ in the addition–elimination one are 2.0 kcal mol^−1^ and 1.1 kcal mol^−1^ higher than the harmonic ones, respectively. However, the anharmonic effect does not change the order of the stability of stationary point structures. It should be noted here that direct treatment of nuclear quantum nature allows us to analyze not only anharmonic correction on electronic energy but also geometry relaxation effect conveniently. So, we would like to revisit these reactions with the aid of our own-developed multicomponent QM (MC_QM)^16–18^ method, which can directly take account of nuclear quantum effect of light nuclei, such as proton and triton, in the near future.

The relative energies corrected for harmonic and anharmonic ZPVE [kcal mol^−1^] of stationary point structures in H-T exchange reactions of C_2_H_4_ with two HTO molecules

**Table:** 

Direct	Reactant	TS_exchange_	Product
Harmonic	0.0	45.5	−0.1
Anharmonic	0.0	47.5	−0.2

**Table:** 

Addition–elimination	Reactant	TS_exchange_	IM_CH_2_OTCH_2_T_	TS_rotation_	IM_CH_2_OTCH_2_T_	TS_elimination_	Product
Harmonic	0.0	36.7	−13.5	−10.1	−13.5	35.6	−0.1
Anharmonic	0.0	37.8	−13.3	−9.3	−13.2	35.9	−0.2

Table 2 shows the relative ZPVE-corrected energies of the reaction of C_2_H_4_ with two HTO molecules obtained in the gas phase and in the solvent models. The SMD solvent model^20^ was used to take into account the solvent effect of water. The relative ZPVE-corrected energies of TS structures in both the direct and the addition–elimination H-T exchange reactions about 7 kcal mol^−1^ raised by including the solvent effect of water. As shown in Table 2, the water solvent effect unstabilized C_2_H_4_ and C_2_H_3_T molecules. On the other hand, HTO molecule was 8.9 kcal mol^−1^ stabilized by the solvent effect. Thus, the total stabilization energy in reactant molecules is −1.0 kcal mol^−1^ + 2 × 8.9 kcal mol^−1^ = 16.8 kcal mol^−1^. Although the TS_exchange_, TS_addition_, and TS_elimination_ were 9.4 kcal mol^−1^, 9.4 kcal mol^−1^, and 9.6 kcal mol^−1^ stabilized by the solvent effect, these stabilization energies were smaller than the aforementioned total stabilization energy in reactant molecules. This is the reason why the relative energies of TS structures become higher in the water solvent environment compared in the gas phase model. The difference between the total stabilization energy in reactant molecule and TS structure is −7.3 kcal mol^−1^ for TS_exchange_, −7.2 kcal mol^−1^ for TS_addition_, and −7.0 kcal mol^−1^ for TS_elimination_. Interestingly, these differences are almost the same to each other, while TS_exchange_ is TS structure of the direct H-T exchange reaction and TS_addition_ and TS_elimination_ are TS structures of the addition–elimination one. The solvent effects in the direct and the addition–elimination H-T exchange reactions are, thus, similar to each other.

The ZPVE-corrected relative energies [kcal mol^−1^] and the ZPVE-corrected total energies [hartree] of stationary point structures in H-T exchange reactions of C_2_H_4_ with two HTO molecules obtained in the gas phase and in the solvent model calculations

**Table:** 

Relative energies			
Direct	Reactant	TS_exchange_	Product
Gas phase	0.0	45.5	−0.1
Solvent model^a^	0.0	52.8	−0.2

a The water solvent effect was taken into account by SCRF(SMD) method.

b Δ*E* is defined as the difference between ZPVE-corrected total energies obtained in the gas phase and in the solvent model calculations.

**Table:** 

Addition–elimination	Reactant	TS_exchange_	IM_CH_2_OTCH_2_T_	TS_rotation_	IM_CH_2_OTCH_2_T_	TS_elimination_	Product
Gas phase	0.0	36.7	−13.5	−10.1	−13.5	35.6	−0.1
Solvent model^a^	0.0	43.9	−11.4	−7.9	−11.4	42.6	−0.2

**Table:** 

Total energies					
	C_2_H_4_	HTO	TS_exchange_	C_2_H_3_T	H_2_O
Gas phase	−78.512289	−76.403392	−231.246610	−78.516634	−76.399268
Solvent model^a^	−78.510640	−76.417519	−231.261556	−78.514983	−76.413449
Δ*E*^b^ [kcal mol^−1^]	1.0	−8.9	−9.4	1.0	−8.9

**Table:** 

	Reactant	TS_exchange_	IM_CH_2_OTCH_2_T_	TS_rotation_	IM_CH_2_OTCH_2_T_
Gas phase	−231.260652	−154.941285	−154.935976	−154.941253	−231.266537
Solvent model^a^	−231.275706	−154.950424	−154.944892	−154.950372	−231.281901
Δ*E*^b^ [kcal mol^−1^]	−9.4	−5.7	−5.6	−5.7	−9.6

It should be noted here that the hybrid-type treatment, in which a few important water molecules in the first solvation-shell are treated explicitly, and the solvation effect from other water molecules are implicitly treated by SCRF method, is widely used and really useful to include the solvent effect efficiently. However, to determine the appropriate number of water molecules required for the reaction is a complicated problem, as Dong previously pointed out.^5^ Our results obtained in the gas phase calculations (Fig. 2) clearly suggest that at least two HTO molecules should be treated in the explicit fashion in both direct and addition–elimination H-T direct exchange reactions, or the reactivity of small organic molecules for H-T exchange reactions with HTO molecules cannot be adequately estimated. Although the implicit treatment of solvent affects the relative energy of TS structures as shown in Table 2, it brings similar unstabilization effect on both the direct and the addition–elimination H-T exchange reactions. Therefore, we believe that the reactivity of small organic molecules for H-T exchange reactions can be adequately evaluated by the gas phase calculations if the second HTO molecule is adequately taken into account.

**Fig. 3:**
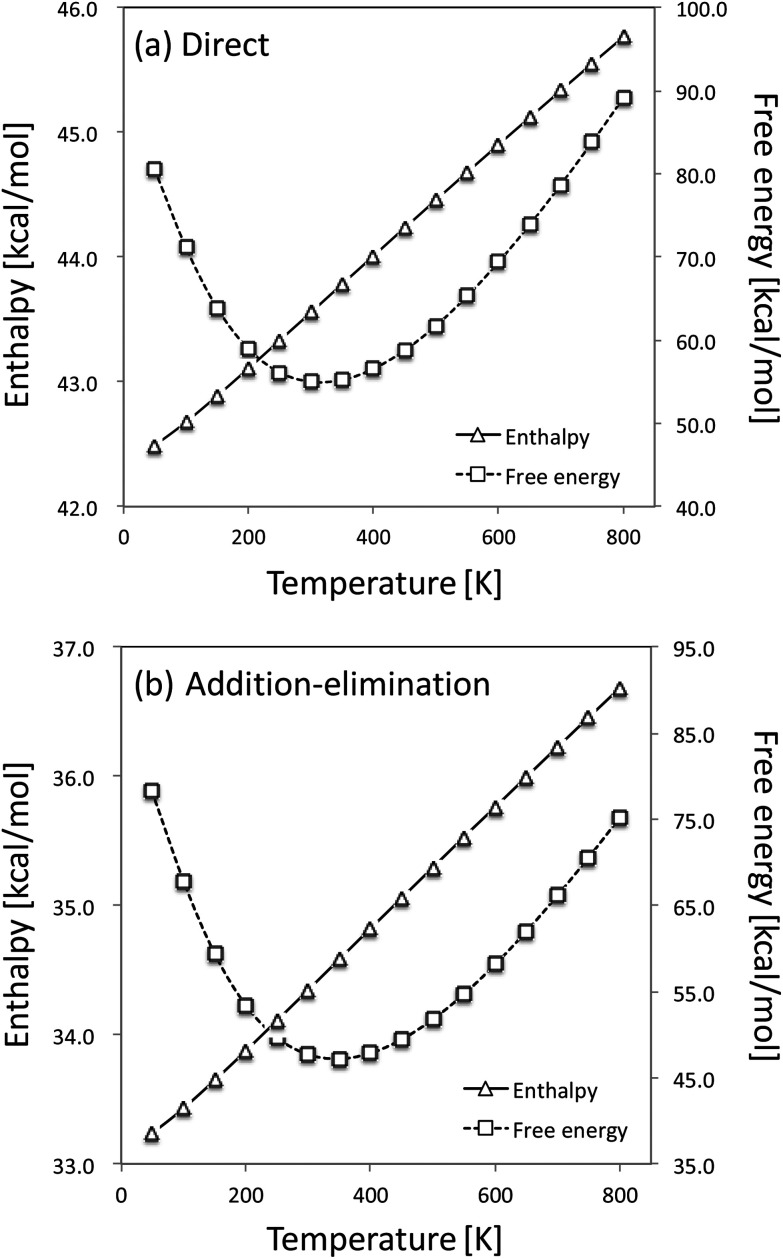
Temperature dependences of the relative enthalpy [kcal mol^−1^] and the relative Gibbs free energy [kcal mol^−1^] in the reaction of C_2_H_4_ with two HTO molecules.

We have also checked the temperature effect on the relative enthalpies and free energies of TS structures in the reaction of C_2_H_4_ with two HTO molecules. Fig. 3 shows the temperature dependence of the calculated relative enthalpies and free energies of TS_exchange_ in the direct H-T exchange mechanism and TS_addition_ in the addition–elimination one. Clearly, the direct and the addition–elimination H-T exchange reactions show similar temperature dependences of enthalpy and free energy. Therefore, we would like to analyze the reaction mechanism of H-T exchange reactions between several organic and HTO molecules by the gas phase calculations and using the thermodynamic properties calculated at the standard conditions (298.15 K, 1 atm) in this study.

To discuss the effect of the second HTO molecule on direct H-T exchange and addition–elimination H-T exchange mechanisms in detail, we applied the activation strain model (ASM) analysis^19^ on ZPVE-corrected energies of activation (Δ*E*^a^) of these reactions. In ASM analysis, the Δ*E*^a^ value is divided into the strain energy (Δ*E*_strain_) and the interaction energy (Δ*E*_int_):

1 Δ*E*^a^ = Δ*E*_strain_ + Δ*E*_int_

The Δ*E*_strain_ energy corresponds to the energy required for the geometrical deformations of the fragments (in the present case, reactant molecules) from the optimized structure of each isolated molecule to the geometry of the TS structure, and the Δ*E*_int_ energy accounts for all chemical interactions. Table 3 lists the Δ*E*_strain_ and the Δ*E*_int_ energies in the reaction of C_2_H_4_ obtained by ASM analysis. We additionally calculated direct H-T exchange reaction of C_2_H_4_ + HTO for comparison. It should be noted here again that only four-membered ring TS structure can be formed in the reaction with one HTO molecule, whereas more stable six-membered ring one can be formed with two HTO molecules.

**Table:** The relative energies for activation (Δ*E*^a^) [kcal mol^−1^], the strain energies (Δ*E*_strain_) [kcal mol^−1^], and the interaction energies (Δ*E*_int_) [kcal mol^−1^] for direct H-T exchange and addition–elimination H-T exchange reactions of C_2_H_4_ with one or two HTO molecules

Reaction	# of HTO	Δ*E*^a^ (ΔΔ*E*^a^^a^)	Δ*E*_strain_ of each fragment			Δ*E*_strain_ (ΔΔ*E*_strain_^a^)	Δ*E*_int_ (ΔΔ*E*_int_^a^)
			C_2_H_4_	HTO(1)	HTO(2)		
Direct	1	71.1	29.8	31.0		60.8	10.3
	2	45.5 (−25.6)	38.8	21.2	17.8	77.8 (17.0)	−32.3 (−42.6)
Addition–elimination	1	54.5	12.5	23.4		35.9	18.5
	2	36.7 (−17.7)	19.0	11.1	19.1	49.1 (13.2)	−12.5 (−30.0)

a ΔΔ*E* is defined as the difference between Δ*E* energies in the reaction with two HTO molecules and with one HTO molecule.

The Δ*E*^a^ value of direct H-T exchange reaction with one HTO molecule is 71.1 kcal mol^−1^, which is much higher than that with two HTO ones (45.5 kcal mol^−1^). Although the sum of Δ*E*_strain_ energies of the reaction with two HTO molecules becomes 17.0 kcal mol^−1^ greater than that with one HTO one by participating the additional (second) HTO molecule to the reaction, the presence of the second HTO molecule makes the orientation of each molecule in TS more suitable to interact with each other, as well as the reaction of CH_4_.^5^ Indeed, Δ*E*_int_ energy in the reaction of C_2_H_4_ with two HTO molecules is much lower than that with one HTO one. The difference between Δ*E*_int_ energies of the direct H-T exchange reactions with one and two HTO molecules (ΔΔ*E*_int_) is −42.6 kcal mol^−1^, and Δ*E*_int_ becomes negative (−32.3 kcal mol^−1^). Hence, Δ*E*_int_ brings the stabilization effect on TS of direct H-T exchange mechanism of the reaction of C_2_H_4_ with two HTO molecules. This large negative ΔΔ*E*_int_ energy overwhelms the unstabilization contribution from ΔΔ*E*_strain_ (17.0 kcal mol^−1^). Consequently, the presence of the second HTO molecule 25.6 kcal mol^−1^ lowers the Δ*E*^a^ value in the reaction of C_2_H_4_. We would like to note here that a non-tritium-substituted H_2_O molecule also may lower the Δ*E*^a^ value of the reaction of C_2_H_4_ with one HTO molecule, since the stable six-membered ring TS structure also can be formed with not only two HTO molecules but also H_2_O and HTO molecules.

Table 3 also lists the results of ASM analysis for addition–elimination H-T exchange reactions of C_2_H_4_ with one or two HTO molecules. First of all, we can find lower Δ*E*^a^ values in addition–elimination reactions rather than in direct exchange ones when the same number of HTO molecules participates to the reaction. For the addition–elimination reaction with one HTO molecule, the Δ*E*_strain_ energy of each fragment is 12.5 kcal mol^−1^ for C_2_H_4_ and 23.4 kcal mol^−1^ for HTO molecule, and the sum of them is 35.9 kcal mol^−1^, which is much smaller than that of direct exchange reaction (60.8 kcal mol^−1^). Thus, in the direct H-T exchange reaction, larger geometrical deformations of both C_2_H_4_ and HTO fragments are required to get their geometries in TS structure rather than in addition–elimination one. Although Δ*E*_strain_ in addition–elimination reaction with one HTO molecule (35.9 kcal mol^−1^) is less than half of that in direct H-T exchange reaction with two HTO ones (77.8 kcal mol^−1^), the Δ*E*^a^ value of addition–elimination H-T exchange reaction of C_2_H_4_ with one HTO molecule is slightly (8.9 kcal mol^−1^) larger than that of direct H-T exchange reaction with two HTO ones. It should be noted here that although the participation of the second HTO molecule lowers the Δ*E*^a^ values, it destabilizes the Δ*G*^a^ values at the same time due to entropic contributions. Consequently, the Δ*G*^a^ values are comparable between direct H-T exchange reactions with two HTO molecules and addition–elimination ones with one HTO molecule for the reactions between alkene and HTO molecules, as discussed above. As in the case of the aforementioned direct H-T exchange reactions, we believe that high-strained four-membered ring TS structure is attributed to the positive Δ*E*_int_ energy in the addition–elimination reaction with one HTO molecule. Thus, we would like to analyze the effect of the second HTO molecule on addition–elimination H-T exchange reactions. The TS of addition process of H-T exchange reaction of C_2_H_4_ can form the more stable six-membered ring TS structure when the second HTO molecule exists, as shown in Fig. 2. The Δ*E*^a^ value is 36.7 kcal mol^−1^ for the addition–elimination reaction of C_2_H_4_ with two HTO molecules. The second HTO molecule, thus, 17.7 kcal mol^−1^ lowers the Δ*E*^a^ energy of the C_2_H_4_ + HTO reaction. To interpret the effect of the second HTO molecule on addition–elimination H-T exchange reaction in detail, Δ*E*_strain_ and Δ*E*_int_ energies are also listed in Table 1. As well as the direct H-T exchange reactions, although the presence of the second HTO molecule brings the destabilization in Δ*E*_strain_ energy (13.2 kcal mol^−1^ of ΔΔ*E*_strain_), it also brings the greater stabilization in Δ*E*_int_ energy (−30.0 kcal mol^−1^ of ΔΔ*E*_int_), which overwhelms the unstabilization contribution from ΔΔ*E*_strain_. Consequently, the second HTO molecule brings the 17.7 kcal mol^−1^ of stabilization in Δ*E*^a^ energy.

The most important point is the fact that the lower Δ*E*^a^ value is found in addition–elimination mechanism rather than in direct H-T exchange one. Unlike the Dong's conclusion,^5^ our results indicate that the addition–elimination H-T exchange mechanism is kinetically favored for the reaction of C_2_H_4_. As shown in Fig. 1 and 2, addition–elimination H-T exchange reactions are favored for not only the reaction of C_2_H_4_ but also the reaction of C_3_H_6_ when the effect of the second HTO molecule is adequately taken into account. Therefore, the second HTO molecule is indispensable to reveal the reaction mechanism of H-T exchange reactions between small organic and HTO molecules.

Although the second HTO molecule also lowers the Δ*E*^a^ values of the reactions of CH_3_CHO, CH_3_COCH_3_, and CH_3_COOH, direct H-T exchange mechanism is still favored for these reactions. The Δ*E*^a^ values in direct H-T exchange reactions are still lower than those in addition–elimination H-T ones when C=O or COOH group participates to the reactions.

Let us summarize the effect of the second HTO molecule on H-T exchange reactions between small organic and HTO molecules. For the direct H-T exchange reactions, our results are consistent with the Dong's conclusion from the ZPVE-corrected energetic point of view,^5^ that is, the reactivity of small organic molecules for direct H-T exchange reaction is in the order of CH_3_COOH (carboxylic acid) > CH_3_CH_2_OH (alcohol) > CH_3_CHO (aldehyde) ≈ CH_3_COCH_3_ (ketone) > C_2_H_4_ and C_3_H_6_ (alkene) > CH_4_, C_2_H_6_, and C_3_H_8_ (alkane). However, we revealed that the second HTO molecule significantly lowered the energies of activation of addition–elimination H-T exchange reactions of alkene by forming a stable six-membered TS structure, although the entropic contribution became more prominent by presence of the second HTO molecule. Consequently, addition–elimination H-T exchange mechanism is favored for the reactions between alkene and two HTO molecules, and the reactivity of small organic molecules for H-T exchange reaction is in the order of CH_3_COOH (carboxylic acid) > CH_3_CH_2_OH (alcohol) > CH_3_CHO (aldehyde) ≈ CH_3_COCH_3_ (ketone) ≈ C_2_H_4_ and C_3_H_6_ (alkene) > CH_4_, C_2_H_6_, and C_3_H_8_ (alkane). Our present study clearly demonstrates the importance of the second HTO molecule for understanding the reaction mechanisms of H-T exchange reactions between small organic and HTO molecules adequately.

In the present study, we used the conventional DFT calculations for the analyses. In the conventional DFT framework, only electronic structures under the field of clamped nuclei are solved based on the Born–Oppenheimer approximation. Thus, the difference between the electronic structures of tritium-non-substituted compound (H-compound) and tritium-substituted compound (T-compound) cannot be represented in the conventional DFT calculations. Only ZPVEs and frequency-dependent properties, such as enthalpies and free energies, differ between H- and T-compounds. On the other hand, we have recently proposed MC_QM methods^16–18^ to directly take account of nuclear quantum effect of hydrogen nuclei. Using MC_QM methods, we can analyze the differences of electronic structures and geometries between H- and T-compounds. We, thus, would like to revisit H-T exchange reactions between small organic and HTO molecules with the aid of MC_QM method in the near future.

## Conclusions

The mechanisms of H-T exchange reactions between small organic and HTO molecules have been investigated using M06-2X/6-311++G(d,p) DFT method. Our study clearly demonstrated that second HTO molecule is also important for addition–elimination H-T exchange reactions. The presence of the second HTO molecule significantly lowers the energies of activation in addition–elimination H-T exchange reactions by participating to form a six-membered ring TS structure, while the presence of the second HTO molecule brings larger entropic contribution at the same time. In particular, the second HTO molecule is especially important for the reactions of alkene. For these reactions, addition–elimination H-T exchange processes are more kinetically favoured than direct H-T exchange ones when the effect of the second HTO molecule is adequately taken into account. Therefore, we can conclude that (i) the reactivity of small organic molecules for H-T exchange reactions with HTO molecule(s) is in the order of CH_3_COOH (carboxylic acid) > CH_3_CH_2_OH (alcohol) > CH_3_CHO (aldehyde) ≈ CH_3_COCH_3_ (ketone) ≈ C_2_H_4_ and C_3_H_6_ (alkene) > CH_4_, C_2_H_6_, and C_3_H_8_ (alkane), and (ii) the H-T exchange reactions between alkene and two HTO molecules occur through addition–elimination H-T exchange mechanism, whereas the reactions between other organic and HTO molecules favour direct H-T exchange mechanism.

## Conflicts of interest

There are no conflicts to declare.

## Supplementary Material

## References

[cit1] Kim S. B., Baglan N., Davis P. A. (2013). J. Environ. Radioact..

[cit2] Takeishi T., Kotoh K., Kawabata Y., Tanaka J., Kawamura S., Iwata M. (2015). Fusion Sci. Technol..

[cit3] Olejniczak A., Fall J., Olejniczak K., Gustova M. V., Shostenko A. G. (2016). J. Radioanal. Nucl. Chem..

[cit4] Magnomedbekov É. P., Shalygin V. A., Baranova O. A., Yu. Isaeva M., Zharkov A. V. (2005). At. Energy.

[cit5] Dong L., Yang N., Yang Y., Li W., Quan Y., Deng B., Meng D., Du Y., Li S., Tan Z. (2017). RSC Adv..

[cit6] Kataoka N., Imaizumi H., Kano N. (2012). Nucl. Sci. Technol..

[cit7] Galeriu D., Melintescu A., Strack S., Atarashi-Andoh M., Kim S. B. (2013). J. Environ. Radioact..

[cit8] Dong L., Xie Y., Du L., Li W., Tan Z. (2015). J. Hazard. Mater..

[cit9] Becke A. D. (1993). J. Chem. Phys..

[cit10] Yanai T., Tew D. P., Handy N. C. (2004). Chem. Phys. Lett..

[cit11] Zhao Y., Truhlar D. G. (2008). Theor. Chem. Acc..

[cit12] Chai J.-D., Head-Gordon M. (2008). Phys. Chem. Chem. Phys..

[cit13] Chai J.-D., Head-Gordon M. (2008). J. Chem. Phys..

[cit14] F Frisch M. J. , Trucks G. W., Schlegel H. B., Scuseria G. E., Robb M. A., Cheeseman J. R., Scalmani G., Barone V., Mennucci B., Petersson G. A., Nakatsuji H., Caricato M., Li X., Hratchian H. P., Izmaylov A. F., Bloino J., Zheng G., Sonnenberg J. L., Hada M., Ehara M., Toyota K., Fukuda R., Hasegawa J., Ishida M., Nakajima T., Honda Y., Kitao O., Nakai H., Vreven T., Montgomery Jr J. A., Peralta J. E., Ogliaro F., Bearpark M., Heyd J. J., Brothers E., Kudin K. N., Staroverov V. N., Keith T., Kobayashi R., Normand J., Raghavachari K., Rendell A., Burant J. C., Iyengar S. S., Tomasi J., Cossi M., Rega N., Millam J. M., Klene M., Knox J. E., Cross J. B., Bakken V., Adamo C., Jaramillo J., Gomperts R., Stratmann R. E., Yazyev O., Austin A. J., Cammi R., Pomelli C., Ochterski J. W., Martin R. L., Morokuma K., Zakrzewski V. G., Voth G. A., Salvador P., Dannenberg J. J., Dapprich S., Daniels A. D., Farkas O., Foresman J. B., Ortiz J. V., Cioslowski J. and Fox D. J., Gaussian 09, Revision B.01, Gaussian, Inc., Wallingford CT, 2010

[cit15] Udagawa T., Tachikawa M. (2009). J. Mol. Struct.: THEOCHEM.

[cit16] Tachikawa M., Mori K., Nakai H., Iguchi K. (1998). Chem. Phys. Lett..

[cit17] Udagawa T., Tachikawa M. (2006). J. Chem. Phys..

[cit18] Udagawa T., Suzuki K., Tachikawa M. (2015). ChemPhysChem.

[cit19] Wolters L. P., Bickelhaupt F. M. (2015). WIREs Comput. Mol. Sci..

[cit20] Marenich A. V., Cramer C. J., Truhlar D. G. (2009). J. Phys. Chem. B.

